# Testing the Immunomodulatory Effects of Probiotic Bacillus coagulans SNZ 1969® in Healthy Adults: A Randomized, Double-Blind, Placebo-Controlled Trial

**DOI:** 10.7759/cureus.94845

**Published:** 2025-10-18

**Authors:** Krishna Murthy D, Raunak J Soman, Dhruv Soman, Kishan PV

**Affiliations:** 1 General Medicine, Vasavi Hospital, Hyderabad, IND; 2 Medical Affairs, Sanzyme Biologics Private Limited, Hyderabad, IND

**Keywords:** bacillus coagulans snz 1969®, immunoglobulin a, natural killer cells, nk cell activity, probiotics

## Abstract

Background: The immune system and the gastrointestinal tract are intricately linked. The intestinal barrier, microbiome, and immune system are in constant communication, shaping immune responses and maintaining homeostasis. Imbalances in the gut microbiome can affect the intestinal barrier and increase susceptibility to infections, along with a decline in immune function (both innate and adaptive immunity). Maintaining optimal immune function is crucial for protecting against infections and supporting overall health, particularly in populations that may be more vulnerable to seasonal respiratory and gastrointestinal infections. Probiotics, particularly spore-forming strains, demonstrate potential for improving natural killer (NK) cell function and mucosal immunity through gut-associated lymphoid tissue interactions. This study evaluated the immunomodulatory effects of *Bacillus coagulans* SNZ 1969^®^ in adults.

Methods: This randomized, double-blind, placebo-controlled clinical trial enrolled adults aged 60-65 years who were susceptible to seasonal infections. Participants were randomized 1:1 to receive either *B. coagulans* SNZ 1969^®^ (2 billion CFU/day) or placebo for 12 weeks. Primary endpoints included NK cell activity, absolute NK cell count (CD3⁻/CD16⁺/CD56⁺), and immunoglobulin levels (serum IgM, IgG, and IgA and salivary IgA). Secondary outcomes assessed respiratory and gastrointestinal infection incidence, inflammatory markers (C-reactive protein), and safety parameters.

Results: Of 60 enrolled participants, 50 completed the study (25 per group). *B. coagulans* SNZ 1969^®^ significantly enhanced NK cell activity compared to placebo, with a net increase of 42.07% between groups (44.59% versus 2.52% increase from baseline; p = 0.0002). NK cell activity improvements were consistent across both genders in exploratory subgroup analyses (limited by small female n = 7-8 per arm; males: 36.75% versus 2.52%; p = 0.00004; females: 63.64% versus 2.71%; p = 0.01155). Significant improvements were observed in serum IgA (25.00% versus 2.30% change; p = 0.0016) and salivary IgA (27.70% versus 0.60% change; p = 0.0002). No significant changes occurred in absolute NK cell counts, serum IgM, IgG, or C-reactive protein levels. Secondary analyses showed numerical reduction trends in upper respiratory tract infections (20% versus 32%; p = 0.11), gastrointestinal infections (8% versus 28%), and total illness days (23 days versus 35 days), favoring the probiotic group, though statistical significance was not achieved. The probiotic was well-tolerated with no serious adverse events.

Conclusions: *B. coagulans* SNZ 1969^®^ supplementation significantly enhanced NK cell activity and mucosal IgA production in adults, suggesting its potential role in strengthening the innate immune defense mechanisms. These findings support the role of *B. coagulans* SNZ 1969^®^ as a safe dietary supplement for augmenting innate cellular immune function and thereby potentially contributing to a reduced trend to infection susceptibility; however, these preliminary findings require more extensive investigation in a larger study population.

## Introduction

The gastrointestinal (GI) tract's relationship with the rest of the body is complex and multifaceted, with the immune system being particularly closely intertwined. The GI tract represents the largest immune organ, housing approximately 70% of immune cells and serving as the primary interface between host and external antigens [1]. The gut-associated lymphoid tissue (GALT) maintains immune homeostasis by orchestrating responses to either mount inflammation against pathogens or induce tolerance toward beneficial entities, such as nutrients and probiotics [2]. This mucosal immune system influences both local and systemic immunity, with natural killer (NK) cells serving as the cornerstone of innate immunity through direct cytotoxicity and cytokine production (e.g., interferon-γ), while immunoglobulin A (IgA) provides barrier defense against pathogens [3].

Modern lifestyle factors including psychological stress, inadequate sleep, reduced physical activity, and ultra-processed diets, collectively compromise the immune function, leading to increased susceptibility to infections, autoimmune disorders, and reduced vaccine efficacy [4]. Decline in the immune competence manifests as reduced NK cell cytotoxicity, impaired T-cell responses, decreased antibody production, and compromised mucosal immunity. NK cells and immunoglobulins work synergistically to provide comprehensive immune protection. NK cells eliminate infected or malignant cells through direct cytotoxicity and release of immunomodulatory cytokines like interferon-γ, which enhances broader immune responses. Simultaneously, IgA antibodies (both in serum and mucosal secretions) form the first line of defense by neutralizing pathogens at mucosal surfaces and preventing their systemic dissemination [5,6]. Elevated serum IgA reflects enhanced systemic immune readiness, while increased salivary IgA strengthens mucosal barrier function. Together, optimized NK cell activity and robust IgA responses create a multilayered defense system that reduces infection risk and supports overall immune resilience [7].

Probiotics are live microorganisms that, when administered in adequate amounts, confer health benefits to the host and are associated with health benefits including modulation of gut microbiota, bolster gut barrier function, and boost overall immunity [8-10]. *Bacillus coagulans* SNZ 1969^®^ is a spore-forming, highly resilient, lactic acid-producing bacterium [11,12]. *B. coagulans* SNZ 1969^®^ is an extensively studied strain and is safe for human consumption, notified as Generally Recognized as Safe (GRAS) vide GRAS Notice (GRN) No. 597 by the United States Food and Drug Administration (USFDA), and *B. coagulans* has European Food and Safety Authority (EFSA) - Qualified Presumption of Safety (QPS) status [13-15].

*B. coagulans* is resilient to gastric acidity and bile acids, ensuring high survivability in the gut and potentially enhancing interactions with GALT [12,16]. This strain has demonstrated promising effects in reducing illness duration in children and improving symptom severity and quality of life in patients with irritable bowel syndrome [11,12]. Clinical evidence on the effect of *B. coagulans* SNZ 1969^®^ on NK cell activity and immunoglobulin dynamics remains limited. Therefore, we evaluated the immunomodulatory effects of *B. coagulans* SNZ 1969^®^ in healthy adults, with a focus on NK cell function and mucosal immunity.

## Materials and methods

Study design and participants

**Figure 1:**
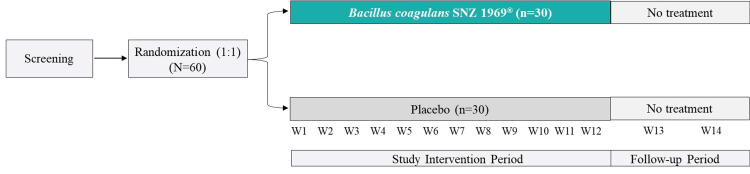
Study design

In this study, Consolidated Standards of Reporting Trials (CONSORT) guidelines were followed. This was a randomized, double-blind, placebo-controlled clinical trial designed to evaluate the immunomodulatory effects of *B. coagulans* SNZ 1969^®^ in healthy adults. The trial was conducted over a 12-week treatment period, followed by a two-week safety follow-up. Participants were randomly assigned in a 1:1 ratio to receive either *B. coagulans* SNZ 1969^®^ or a placebo (Figure 1).

Inclusion criteria included healthy male and female adults aged 60-65 years who were clinically stable with no chronic diseases (e.g., hypertension, diabetes, cardiovascular disease, or rheumatoid arthritis), susceptible to seasonal infections defined as self-reported history of ≥2 episodes of upper respiratory tract infections (URTIs) or GI tract infections (GITIs) (e.g., colds, coughs, fevers, and diarrhea) in the previous year, confirmed by investigator medical history review, with baseline NK cell activity ≤ 15%, and with willingness to comply with study requirements and provide written informed consent. The 60-65-year age group was chosen to capture subtle immune declines associated with early aging. The NK activity cutoff ≤ 15% was selected to identify participants with mildly impaired NK function consistent with early aging, as NK activity in elderly adults is typically 10%-30% lower than in young adults (e.g., 10%-15% in Calcein AM assays) compared to younger adults [15]. Exclusion criteria comprised the use of immunosuppressive therapy, recent antibiotic or probiotic use within four weeks prior to enrolment, chronic illness requiring ongoing treatment, participation in another clinical trial within 30 days, and allergy or intolerance to study product components.

Study treatments and the supplementation procedure

*B. coagulans* SNZ 1969^®^ is a rod-shaped, slightly acidophilic, gram-positive, spore-forming, highly resilient, lactic acid-producing bacterium. Participants were administered capsules containing either *B. coagulans* SNZ 1969^®^ (2 billion CFU/capsule; Sanzyme Biologics Private Limited, Hyderabad, India) or placebo (maltodextrin) orally with water once daily for 12 weeks. Capsules were identical in appearance, taste, and packaging to ensure blinding.

The investigational product (*B. coagulans* SNZ 1969^®^) and an identical placebo were supplied by Sanzyme Biologics Private Limited. Randomization was performed using computer-generated codes, and double-blinding was ensured by independent labeling of the intervention and placebo. Participants were instructed to take the capsule at the same time each day. Use of probiotics, antibiotics, or immunomodulatory drugs was prohibited during the study, and the use of any concomitant medications was reported.

Study procedures and outcome measures

Peripheral blood samples were collected at baseline (Visit 1) and Week 12 (Visit 2) to assess primary endpoints. Primary outcomes included changes from baseline to Week 12 in NK cell activity, measured using a fluorescence-based cytotoxicity assay (Calcein AM) against K562 target cells with results expressed as percentage lysis relative to baseline; absolute NK cell counts determined via flow cytometry targeting CD3^-^/CD16^+^/CD56^+^ cells; and immunoglobulin levels, where serum IgM, IgG, and IgA and salivary IgA were analyzed using enzyme-linked immunosorbent assays. Secondary outcomes assessed changes from baseline to Week 12 in the infection incidence and duration, with URTI and GITI infections recorded in patient diaries and verified by investigators; inflammatory markers, specifically serum C-reactive protein (CRP), measured using immunoturbidimetry; and emergency medical visits and total illness days.

Secondary outcomes were monitored throughout the 12-week period using patient diaries and clinical evaluations. Adverse events (AEs) were recorded at each visit, and treatment compliance was assessed via capsule counts and patient diaries.

Method of NK cell activity assay

The standard assay for NK cell activity involved K562 cells (1 × 10⁶/mL) incubated with Calcein AM dye (16 μM) at 37°C for 30 minutes. After incubation, the cells were centrifuged, washed with phosphate buffer solution (PBS), and further incubated for four hours at 37°C with 5% CO₂ in the presence of effector cells (peripheral blood mononuclear cells) at a 1:50 ratio (10⁴ K562 cells: 5 × 10⁵ lymphocytes), while K562 cells alone served as the control. Following this, the cells were centrifuged, washed twice with PBS, and suspended in 100 μL PBS. Fluorescence measurements were recorded at λₑₓ = 495 nm and λₑₘ = 530 nm, and NK cell lytic activity (%) was calculated using the following formula [17]. The formula was verified for consistency across samples:

$(F_{C} - F_{T}) \times 100 / F_{C}$

where *F_C_* is the intensity for control K562 and *F_T_* is the intensity for K562 in the presence of NK cells.

Statistical analysis

The sample size calculation was based on the study’s primary endpoint: change in NK cell activity from baseline to Week 12, measured using a fluorescence-based Calcein AM cytotoxicity assay against K562 target cells. Estimates for effect size were guided by previously published clinical studies investigating the modulation of NK cell activity by probiotic supplementation in healthy adults [18]. Anticipating a between-group difference of 30% in NK cell activity and a standard deviation (SD) of 20%, a two-sided significance level of 0.05, and 80% power (1 - β = 0.80), a sample size of 25 subjects per group was determined to be necessary to detect this difference [19]. Accounting for a possible dropout rate of 20%, the total recruitment goal was set at 30 participants per group (N = 60). Sample size calculations were performed using G*Power version 3.1.9.7 (Heinrich-Heine-Universität Düsseldorf, Düsseldorf, Germany) [20]. Analyses were conducted using GraphPad Prism version 9.0 (GraphPad Software, Dotmatics, Boston, MA, US).

The full analysis set (FAS) included all randomized participants who received at least one dose of the study intervention. All the analyses were performed for the changes from baseline to Week 12. Descriptive statistics (mean, SD, and range) were calculated for continuous variables, and frequency counts and percentages were used for categorical variables. Normality was assessed using the Shapiro-Wilk test. For normally distributed data, independent t-tests were applied; for non-normal data, Mann-Whitney U tests were used. Incidence rates of infections were compared using Chi-squared or Fisher’s exact tests. A p-value < 0.05 was considered statistically significant.

## Results

**Figure 2:**
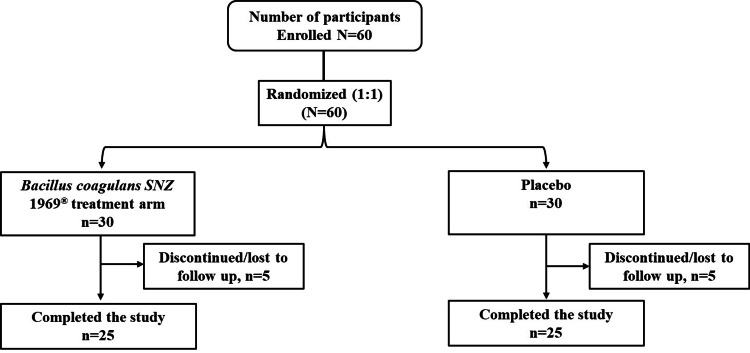
CONSORT flow chart CONSORT: Consolidated Standards of Reporting Trials

A total of 60 participants were screened, enrolled, and randomized 30 per arm, to receive either *B. coagulans* SNZ 1969^®^ (n = 30) or placebo (n = 30). Ten participants discontinued the study (five in each group due to withdrawal or loss to follow-up), with 50 completing the study and analyzed in the FAS (Figure 2). The mean (SD) age of participants in the probiotic *B. coagulans* SNZ 1969^®^ arm was 62.28 (1.9) years, compared to 62.68 (1.84) years in the placebo group. The age range across both groups was 60-65 years, with men comprising 68% of the probiotic group and 72% of the placebo group (Table 1). Baseline characteristics, including NK cell activity, NK cell count, and immunoglobulin levels, were comparable between groups (Table 2). Treatment compliance was 100%, as assessed by capsule counts and patient diaries. No protocol deviations were reported.

**Table 1:** Baseline demographics and clinical characteristics SD: standard deviation

Characteristic	*Bacillus coagulans *SNZ 1969^®^ (n = 25)	Placebo (n = 25)
Age (years)		
Mean ± SD	62.28 ± 1.90	62.68 ± 1.84
Sex, n (%)		
Male	17 (68.00)	18 (72.00)

**Table 2:** Summary of primary endpoints Data are presented as mean ± SD. p-values reflect comparisons of percent change from baseline to Week 12 using ^a^Mann-Whitney U test or ^b^t-test based on data normality *p < 0.05 is for *B. coagulans* SNZ 1969^®^ vs. placebo CD: cluster of differentiation; IgA/M/G: immunoglobulin A/M/G; NK cells: natural killer cells; SD: standard deviation

Parameter	*Bacillus coagulans* SNZ 1969^®^ (n = 25)	Placebo (n = 25)	p-value
NK cell activity analysis (%)			
Baseline	10.53 ± 1.58	10.71 ± 1.54	-
Week 12	15.23 ± 3.02	10.98 ± 2.15	-
Percent change	44.59 ± 28.65	2.52 ± 16.24	0.0002^a^
NK cell activity (%) - males			
Baseline	10.91 ± 1.35	10.72 ± 1.56	
Week 12	14.92 ± 2.78	10.99 ± 1.88	
Percent change	36.75 ± 25.50	2.52 ± 17.50	0.00004^a^
NK cell activity (%) - females			
Baseline	9.71 ± 1.82	10.67 ± 1.58	-
Week 12	15.89 ± 3.59	10.96 ± 2.90	-
Percent change	63.64 ± 37.00	2.71 ± 27.20	0.01155^a^
NK cell absolute count analysis (CD3⁻/CD16⁺/CD56⁺) cells/μL			
Baseline	535.20 ± 141.20	540.40 ± 102.85	-
Week 12	587.24 ± 194.93	553.92 ± 109.34	-
Percent change	9.72 ± 26.47	2.50 ± 16.24	0.4606^b^
Serum IgA (mg/dL)			
Baseline	156.15 ± 54.75	154.23 ± 51.92	-
Week 12	195.16 ± 84.92	157.84 ± 50.50	-
Percent change	25.00 ± 64.70	2.30 ± 46.90	0.0016^a^
Salivary IgA (mg/dL)			
Baseline	11.12 ± 3.26	10.87 ± 2.29	-
Week 12	13.91 ± 3.19	10.84 ± 2.04	-
Percent change	27.70 ± 16.00	0.60 ± 8.00	0.0002^b^
Serum IgM (mg/dL)			
Baseline	143.52 ± 52.82	142.28 ± 75.47	-
Week 12	149.85 ± 51.99	144.54 ± 70.10	-
Percent change	4.17 ± 51.63	1.59 ± 53.20	0.2252^a^
Serum IgG (mg/dL)			
Baseline	1,146.96 ± 321.75	1,144.61 ± 135.49	-
Week 12	1,194.50 ± 287.45	1,122.27 ± 134.44	-
Percent change	4.1 ± 37.70	−1.9 ± 16.70	0.6414^a^

Primary efficacy outcomes

NK Cell Activity

*B. coagulans* SNZ 1969^®^ supplementation resulted in significant enhancement of NK cell cytotoxic activity compared with placebo. Baseline NK cell activity levels were comparable between groups (probiotic: 10.53 ± 1.58%; placebo: 10.71 ± 1.54%). Following 12 weeks of intervention, the probiotic group exhibited a substantial increase in NK cell activity to 15.23 ± 3.02%, representing a 44.59% improvement from baseline, while the placebo group remained essentially unchanged at 10.98 ± 2.15% (2.52% change from baseline; p = 0.0002; Table 2).

Subgroup analysis by sex revealed consistent findings. Among men, baseline NK cell activity was comparable (probiotic: 10.91 ± 1.35%; placebo: 10.72 ± 1.56%). At Week 12, the probiotic group showed a significant increase (14.92 ± 2.78%) compared to the placebo group (10.99 ± 1.88%), with a mean percent change of 36.75% versus 2.52% (p = 0.00004, Mann-Whitney U test). Among women, baseline values were similar (probiotic: 9.71 ± 1.82%; placebo: 10.67 ± 1.58%). By Week 12, the probiotic group increased to 15.89 ± 3.59% compared to 10.96 ± 2.90% in the placebo group, with a mean percent change from baseline of 63.64% versus 2.71% (p = 0.01155, Mann-Whitney U test; Table 2). These sex-stratified results are exploratory and should be interpreted cautiously due to small subgroup sizes and limited statistical power; confirmation in larger cohorts is needed.

NK Cell Counts

Absolute NK cell counts (CD3⁻/CD16⁺/CD56⁺) showed increases in both treatment groups without achieving statistical significance. Baseline counts were equivalent between groups (probiotic: 535.20 ± 141.20 cells/μL; placebo: 540.40 ± 102.85 cells/μL). At Week 12, both groups exhibited increases (probiotic: 587.24 ± 194.93 cells/μL; placebo: 553.92 ± 109.34 cells/μL), with no significant between-group difference in percentage change (9.72% versus 2.50%; p = 0.4606; Table 2).

Immunoglobulin Levels

Serum IgA concentrations increased significantly in the probiotic group compared with placebo. From baseline levels of 156.15 ± 54.75 mg/dL and 154.23 ± 51.92 mg/dL in the probiotic and placebo groups, respectively, Week 12 concentrations reached 195.16 ± 84.92 mg/dL in the probiotic group versus 157.84 ± 50.50 mg/dL in placebo, representing percentage changes of 25.00% and 2.30%, respectively (p = 0.0016).

Salivary IgA exhibited an even more pronounced treatment effect. Baseline concentrations were similar between groups (probiotic: 11.12 ± 3.26 mg/dL; placebo: 10.87 ± 2.29 mg/dL). After 12 weeks, the probiotic group achieved salivary IgA levels of 13.91 ± 3.19 mg/dL compared with 10.84 ± 2.04 mg/dL in placebo, corresponding to percentage changes of 27.70% and 0.60%, respectively (p = 0.0002; Table 2).

Neither serum IgM nor IgG concentrations differed significantly between treatment groups. Both immunoglobulin classes exhibited modest, non-significant increases in both groups (IgM: 4.17% versus 1.59%, p = 0.2252; IgG: 4.10% versus −1.90%, p = 0.6414; Table 2).

Secondary efficacy outcomes

C-Reactive Protein

CRP concentrations were similar at baseline (probiotic: 7.28 ± 2.65 mg/L; placebo: 7.15 ± 1.82 mg/L). At Week 12, the probiotic group exhibited a modest decrease to 7.12 ± 4.23 mg/L while the placebo group increased slightly to 7.57 ± 2.10 mg/L, though this difference was not statistically significant (p = 0.25) (Table 3).

**Table 3:** Summary of secondary endpoints Data are presented as mean ± SD or n (%) *p-values reflect comparisons using the Mann-Whitney U test due to non-normal data distribution CRP: C-reactive protein; GITI: gastrointestinal tract infections; SD: standard deviation; URTI: upper respiratory tract infections

Parameter	*Bacillus coagulans* SNZ 1969^®^ (n = 25)	Placebo (n = 25)	p-value*
CRP (mg/L)			
Baseline	7.28 ± 2.65	7.15 ± 1.82	-
Week 12	7.12 ± 4.23	7.57 ± 2.10	0.25
URTI incidence at Week 12			
n (%)	5 (20.0%)	8 (32.0%)	0.11
Incidence of infections	1.00 ± 0.0	1.33 ± 0.21	-
URTI duration (days)	3.40 ± 0.50	3.33 ± 0.82	1.0
Total duration (days)	17	20	-
GITI incidence			
n (%)	2 (8%)	7 (28%)	0.39
Incidence of infections	1.0 ± 0.0	1.17 ± 0.41	-
Total infection days	2	7	-
GITI duration (days)			
Incidence of infections	3.00 ± 0.00	2.50 ± 0.84	0.36
Total duration (days)	6	15	-
Total days of illness			
Incidence of infections	3.29 ± 0.49	3.89 ± 1.36	0.39
Total days	23	35	-
Emergency medical visits			
n (%)	2 (8.00%)	3 (12.00%)	1.00
Incidence of infections	1.00 ± 0.00	1.00 ± 0.00	-

Infection Incidence and Duration

The incidence of URTIs was 20% (5/25) in the probiotic group and 32% (8/25) in the placebo group (p = 0.11, Mann-Whitney U test). Total URTI days were 17 and 20, respectively, with similar mean durations (probiotic: 3.40 ± 0.50 days; placebo: 3.33 ± 0.82 days; p = 1.0). GITIs occurred in 8% (2/25) of the probiotic group and 28% (7/25) of the placebo group (p = 0.39), with total infection days of six and 15, respectively. Mean GITI duration was 3.00 ± 0.00 days in the probiotic group and 2.50 ± 0.84 days in the placebo group (p = 0.36; Table 3).

Total Days of Illness and Emergency Medical Visits

The mean total days of illness were 3.29 ± 0.49 days (total: 23 days) in the probiotic group and 3.89 ± 1.36 days (total: 35 days) in the placebo group (p = 0.39, Mann-Whitney U test). Emergency medical visits occurred in 8.00% (2/25) of the probiotic group and 12.00% (3/25) of the placebo group (p = 1.00; Table 3).

Safety

No AEs (treatment-related or unrelated), serious AEs, or deaths were reported during the study or two-week follow-up, as monitored via patient diaries and clinical visits.

## Discussion

This study provides new evidence that *B. coagulans* SNZ 1969^®^ enhances immune function in healthy adults aged between 60 and 65 years. The significant increase in NK cell activity (p < 0.05) across men and women demonstrates the potential of *B. coagulans* SNZ 1969^®^ to restore immune surveillance capacity [21,22].

Our findings are consistent with previous research on *B. coagulans* strains [23,24]. For instance, Upadhyaya and Banerjee demonstrated similar NK cell activity enhancement in immune-compromised adults following eight weeks of *B. coagulans* supplementation [24]. Along the same lines, our study findings demonstrate enhanced NK cell activity in healthy individuals rather than immune-compromised individuals, indicating broader and extensive applicability of *B. coagulans* SNZ 1969^®^ for immune support in adult populations [14].

Additionally, *Heyndrickxia coagulans* strain SANK70258, a closely related spore-forming bacterium, has shown comparable immunomodulatory effects with added benefits for URTI prevention. The mechanistic insights from this study suggest that spore-forming probiotics may activate plasmacytoid dendritic cells expressing toll-like receptor 7 and CD304, leading to enhanced IFN-α production and subsequent NK cell activation. This proposed mechanism aligns with our observed NK cell enhancement and provides a potential explanation for the functional improvements we observed through gut-immune axis modulation [25].

The significant elevations in both serum and salivary IgA further support the immunomodulatory effects of *B. coagulans* SNZ 1969^®^. IgA is essential for mucosal defense, preventing pathogen adhesion at respiratory and GI interfaces [26,27]. Our findings are consistent with preclinical data showing *B. coagulans* strains increase intestinal IgA via dendritic cell activation and IL-12/IL-23 pathways [28,29]. Enhanced mucosal immunity may explain the trends in reduction of GI infections (8.0% versus 24.0%, p = 0.39) observed in the probiotic group, although statistical significance was not achieved.

Baseline CRP levels (~7 mg/L) suggest mild low-grade inflammation typical of early aging in this population, without underlying conditions. The absence of significant changes in CRP levels indicates limited impact on systemic inflammation. This is desirable as it demonstrates that *B. coagulans* SNZ 1969^®^ enhances immune function without triggering unwanted inflammatory responses. This finding supports the safety profile of the probiotic intervention, showing that immune enhancement occurs through targeted modulation rather than broad inflammatory activation. Maintaining stable CRP levels while improving NK cell activity and immunoglobulin responses indicates a balanced immunomodulatory effect that strengthens host defense mechanisms without promoting systemic inflammation.

Secondary outcomes, including infection incidence, duration, and total illness days, showed trends favoring the probiotic group; however, they did not reach statistical significance. The lower incidence of GI infections and total illness days in the probiotic group aligns with prior studies on *B. coagulans* reducing GI symptoms [11].

The high compliance rate (100%) and absence of serious AEs underscore the safety of *B. coagulans* SNZ 1969^®^, consistent with its established safety profile. Additionally, the spore-forming nature of this probiotic and gastric acid and bile resistance offer distinct advantages over non-spore-forming probiotic strains, demonstrating efficacy with statistically significant results across diverse populations regardless of gender. This broad applicability suggests preliminary potential for applicability in adult populations [30]. Unlike traditional lactobacilli and bifidobacteria, spore-forming probiotics demonstrate superior gastric acid resistance, bile resistance, and shelf stability, potentially ensuring more consistent delivery of viable organisms to the intestinal tract where they can interact with GALT.

While NK and IgA enhancements suggest immune strengthening, these did not translate to significant infection reductions, possibly due to underpowering (small n = 50), 12-week duration missing seasonal peaks, or the population's relatively mild baseline risk. Additional limitations include a narrow population (60-65 years, 70% male), limiting generalizability, a lack of gut microbiota analysis to support GALT mechanisms, and insufficient power for secondary outcomes. Larger trials are needed to link immunological to clinical outcomes.

## Conclusions

This study demonstrates that supplementation with *B. coagulans* SNZ 1969^®^ significantly enhances immune function, as evidenced by increased NK cell activity, serum IgA, and salivary IgA levels in healthy adults. Additionally, supplementation was associated with a reduced incidence and shorter duration of infections, particularly those affecting the GI and respiratory systems, as well as a decrease in overall illness days. These findings underscore the role of *B. coagulans* SNZ 1969^®^ in strengthening innate immune defense mechanisms and reducing infection susceptibility among adults.

From a safety perspective, *B. coagulans* SNZ 1969^®^ was well-tolerated, with no serious AEs reported. These findings support the role of *B. coagulans* SNZ 1969^®^ as a safe dietary supplement for augmenting innate cellular immune function and thereby reducing infection susceptibility and confer general well-being. These preliminary findings are limited by a small sample size and short duration. Future studies with larger, more diverse populations (e.g., broader ages and balanced sexes), longer durations, and mechanistic analyses (e.g., gut microbiota profiling) are warranted to validate these findings.
